# Seroprevalence and Risk Analysis of *Toxoplasma Gondii* in Wild Birds of District Lahore Punjab, Pakistan

**DOI:** 10.1002/vms3.70907

**Published:** 2026-04-10

**Authors:** Shahzad Ali, Fareeha Imran, Abigail A. Lilak, Tooba Latif, Michael E. von Fricken, Muhammad Tayyub, Usama Saeed, Anam Iftikhar

**Affiliations:** ^1^ Wildlife Epidemiology and Molecular Microbiology Laboratory (One Health Research Group) Discipline of Zoology Department of Wildlife & Ecology University of Veterinary and Animal Sciences Pattoki Pakistan; ^2^ One Health Center of Excellence College of Public Health and Health Professions University of Florida Gainesville Florida USA; ^3^ IIIinois Natural History Survey‐Prairie Research Institute University of Illinois at Urbana‐Champaign Champaign Illinois USA; ^4^ Department of Global and Community Health College of Public Health George Mason University Fairfax Virginia USA; ^5^ Department of Biology University of Okara Okara Pakistan; ^6^ Department of Biological Sciences University of Veterinary and Animal Sciences Pattoki Pakistan

**Keywords:** risk assessment, seroepidemiology, wildlife disease surveillance, zoonotic parasites

## Abstract

**Introduction:**

Toxoplasmosis is the most prevalent zoonotic disease caused by *Toxoplasma gondii*, affecting warm‐blooded species such as birds, humans, and marine mammals. Wild birds contribute significantly to the spread of *T. gondii* through shared contact with other living organisms. Moreover, the consumption of wild bird meat is a potential source of infections in humans. This study aims to determine the seroprevalence of *T. gondii* antibodies in wild birds in the Lahore district of Punjab, Pakistan.

**Methods:**

One hundred and seventy‐six wild birds of 35 species were captured for blood collection from Lahore, Punjab, Pakistan. Data related to demography and epidemiology were collected on field proforma. Serum samples were tested for *T. gondii* antibodies using the latex agglutination test (LAT).

**Results:**

A total of 18 birds, representing 10.2% of the sample, tested positive for *T. gondii* antibodies. Of the bird species sampled, 40% (*n* = 14) of bird species were found seropositive for *T. gondii*. Red‐wattled Lapwing and Indian silver bill had the highest prevalences (50%). A substantial difference (*p* ≤ 0.05) in antibodies of *T. gondii* prevalence was found between adults (28.6%) and juveniles (7.7%). birds, with no differences noted in other parameters based on chi‐square analysis. However, species, age, and locality were identified as potential risk variables for seropositivity of *T. gondii* based on binary logistic regression.

**Discussion:**

This study presents evidence of widespread of *T. gondii* antibodies in wild birds captured in Lahore district. The findings will aid in developing effective strategies to control this disease in the study area.

**Impacts:**

- This study found that over 10% of wild birds in Lahore, Pakistan, carry antibodies for *Toxoplasma gondii*, a parasite that can cause serious illness in humans and animals.
- Birds like lapwings and silver bills showed especially high antibody rates.
- Improved monitoring is needed to better understand risk dynamics in Pakistan.

## Graphical abstract

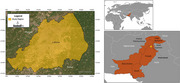

## 1 Introduction

Toxoplasmosis, a zoonotic disease caused by *Toxoplasma gondii*, is highly prevalent due to its ability to infect avian, marsupial, and mammalian taxa. Globally, the prevalence of toxoplasmosis varied greatly. The prevalence rate was reported nearly 90% in some endemic areas of Africa, while up to 60% prevalence was documented in some European countries (Robert‐Gangneux and Dardé 2012). Of note, mortality related to toxoplasmosis is significantly higher in immunocompromised individuals due to reactivation of latent *Toxoplasma gondii* infection in patients with advanced HIV/AIDS, where toxoplasmic encephalitis is a major cause of death (Flegr et al. 2014). Infections are facilitated by the consumption of food and water contaminated with sporulated *T. gondii* oocysts. Felids are hosts of *T. gondii*; they become infected through the consumption of infected tissues of intermediate hosts (Tenter et al. 2000). In addition, *T. gondii* has been detected in water sources used for drinking and recreational activities. Previous studies have identified water contaminated by sewage as the primary source of *T. gondii* oocysts (Aguirre et al. 2019; Shapiro et al. 2014).

*T. gondii* infections are prevalent among poultry, wild birds, and domestic birds. There have been reported clinical cases demonstrating that *T. gondii* can cause mortality in bird species (Dubey 2010; Rosenberg et al. 2019). Like felids, the typical contamination route for birds is consuming contaminated food and water. Vertical transmission of *T. gondii* has not been well documented in bird species but remains not fully understood with current studies and evidence (Mancianti et al. 2013; Rego et al. 2018). In the context of *T. gondii*, birds are an important intermediate host due to their ability for high dispersal while also serving as a proxy for environmental contamination, especially in non‐migratory bird species (Boughattas and Bouratbine 2015; Naveed et al. 2019; Wilson et al., 2020). Scavenger birds, which feed on available resources in urban and peri‐urban settings, have often been studied to further recognise their role in the epidemiology of *T. gondii* infection (Gondim et al. 2010). Potential zoonotic transmission of *T. gondii* may be associated with diets containing meat from birds and improper handling of contaminated meat (Vieira et al. 2018). This transmission route is particularly concerning in regions where wild birds are hunted or consumed without adequate food safety practices.

In addition to felids and birds, *T. gondii* has been found in other species, such as the Arctic fox (*Alopex lagopus*) and polar bear (*Ursus maritimus*) in Norway (Sandstrom et al. 2013). A previous study reported a 79% prevalence of toxoplasmosis in common buzzards (*Buteo buteo*) across Europe, while 80.5% prevalence was reported in common ravens (*Corvus corax*) in Spain (Aubert et al. 2008; Molina et al. 2011). In Pakistan, a recent study focused on toxoplasmosis in wild birds, documented multiple wild birds positive for *T. gondii* antibodies and confirmed toxoplasmosis in sheep and goats (Ahmed et al. 2016; Naveed et al. 2019). Although previous work identifies *T.gondii* likely circulating within multiple species, there remains a significant gap in epidemiological data on *T. gondii* infection in wild bird populations specifically within Pakistan given that most studies focus solely on domestic animals or poultry. This study aimed to determine the seroprevalence of *T. gondii* antibodies in wild birds in the Lahore region of Pakistan and to identify associated ecological and behavioural risk factors.

## 2 Materials and Methods

### 2.1 Study Area

**FIGURE 1:**
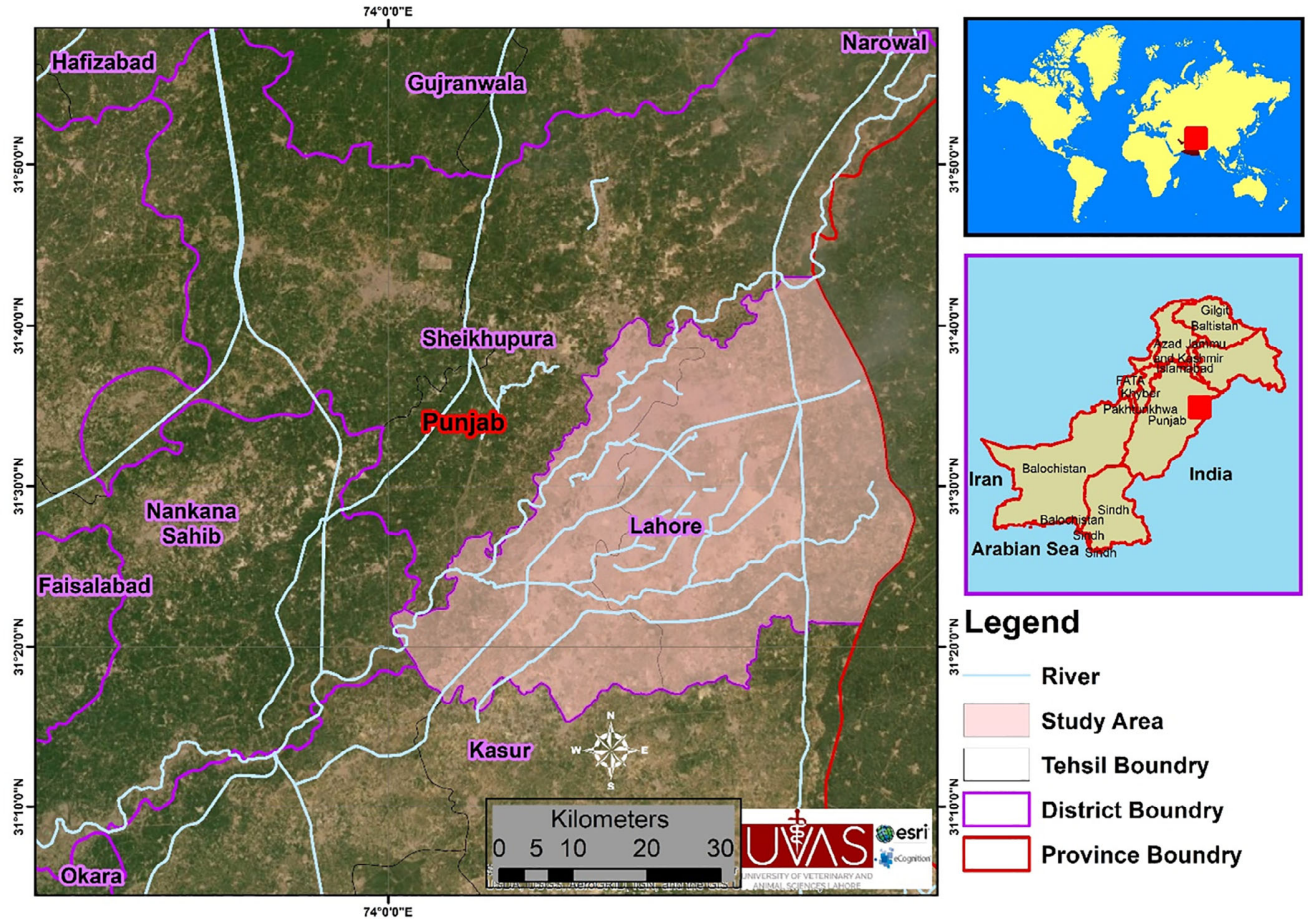
Study area map for sample collection from wild birds from district Lahore, Punjab Pakistan.

The study was conducted in Lahore District, Punjab, Pakistan (31.5204°N, 74.3587°E) as shown in Figure 1. Shalimar, Model Town, Lahore City, Lahore Cantt and Raiwind are five tehsils (administrative divisions) in Lahore District. Lahore covers an area of 1772 km^2^ and serves as the capital of Punjab province, being the second most populous city in Pakistan. Within Lahore, there is a mix of rural and urban landscapes, with a wide range of avian species. Given the diverse habitats located within Lahore, it supports high levels of avian diversity, with previous work identifying 77 species (Iqbal et al. 2007; Mehmood et al. 2018).

### 2.2 Sample and Data Collection

A total of 176 common wild birds from 35 different bird species, classified as least concern by the International Union for Conservation of Nature (IUCN), were identified using a standard key (Grimmett et al. 2008). Once birds were captured, the age and sex was determined using morphological and biometric criteria (Grewal 2017; Grimmett et al. 2008; Svensson 1992). Using standardised ornithological protocols, determination focused on plumage characteristics when there are sexual dimorphism or age‐specific traits, biometric measurements of wing length, tarsus length and body mass to assist with sex in size dimorphic species and cloacal protuberance and brood path during breeding season. For captured species lacking clear dimorphism, age and sex were recorded as undetermined and as a result, were excluded from the analysis and study. Birds were captured using mist netting to ensure safe handling and handling time was minimised for animal welfare. Blood was drawn from the basilica vein of each captured bird using a sterile syringe and transferred into non‐EDTA tubes. Sera was then extracted from the blood samples after centrifugation at 12,000 rpm for 2 min and preserved at –20°C for further analysis (Wilson et al. 2020). All captured birds were released immediately after sample collection and clinical observation. To avoid resampling birds, each sampling session had a single capture, and mist nets were removed after the sampling session (Bibby 2000; Friend et al. 1999).

Demographic data (district and location) and risk variables (bird species, gender, urbanicity, health status, age and residential status) were collected on field proforma. Health status was assessed by observing clinical signs such as lethargy, abnormal posture, ruffled feathers, visible respiratory distress, diarrhoea, ocular abnormalities and any injuries or impaired mobility (Friend et al. 1999). Birds which appeared to be healthy, alert and lacking and of the clinical observations were considered apparently healthy. Urbanicity was based on human population density, infrastructure, vegetation cover and proximity to agricultural land. Respectively, sampling sites were categorised into the following: urban, peri‐urban or rural following urban ecology frameworks (Martin 1976; Ompad et al. 2007). Species which were captured at the sampling sites were classified into the categories of urbanicity based on where it was solely captured and not based on species habitat preference.

### 2.3 Serological Testing

The LAT was employed to screen for *T. gondii* antibodies in serum samples using the Antec Diagnostics assay (Bridport Dorset, UK). Initially, serum samples were diluted in phosphate‐buffered saline (PBS) at ratios of 1:2 to 1:8. Double dilutions were prepared for all positive samples at various ratios such as 1:16, 1:32, and 1:64 per manufacturer guidelines. A sample was termed positive if it contained 4 IU/mL or more of *T. gondii* antibodies; otherwise, it was considered negative. The ultimate titre was determined in these instances. The test's sensitivity was 3–7 IU/mL, much lower in adults within normal ranges.

### 2.4 Mapping

Geographic visualisations were created using ArcGIS Pro 2.7.3 (Esri) and country shapefiles were taken from the Database of Global Administrative Areas (GADM, version 4.0, https://gadm.org/).

### 2.5 Statistical Analysis

Standard descriptive statistics were employed for analysis. Prevalence was estimated using chi‐square test, then followed by a binary logistic regression to evaluate the relationship between seropositivity and explanatory factors. A *p*‐value of ≤ 0.05 indicates a statistically significant variance between the outcome and explanatory factors. This research was completed using the Statistical Package for Social Sciences (SPSS), Version 23.0 (Saeed et al. 2019).

## 3 Results

Eighteen (10.2%) of the 176 serum samples tested positive for *T. gondii* antibodies using LAT. Furthermore, 14 of 35 bird species captured in this study tested positive *T. gondii* antibodies (Table 1). Wild birds tested positive at 14 (56%) sites; however, site‐wise seroprevalence among these sites was not statistically significant (*p* > 0.05) (Table 2). Male birds show a seroprevalence rate of 13.5%, whereas female birds exhibit a rate of 6.9%, but was not statistically significant (*p* > 0.05). Birds from rural areas showed a higher seroprevalence (11.3%) compared to urban areas (8.9%), but the difference was not statistically significant.

**TABLE 1:** Prevalence of *Toxoplasma gondii* in birds from the wild from district Lahore Punjab Pakistan (cut‐off value: 1:64).

Species	Scientific name	Total examined	Infected	Prevalence (%)
Brown rock chat	*Cercomela fusca*	9	2	22.2
House sparrow	*Passer domesticus*	7	0	0
Common myna	*Acridotheres tristis*	3	1	33.3
House crow	*Corvus splendens*	15	3	20
Jungle babbler	*Turdoides striatus*	9	1	11.1
Asian pied starling	*Sturnus contra*	6	1	16.7
Common quail	*Coturnix coturnix*	3	0	0
Red vented bulbul	*Pycnonotus cafer*	10	0	0
Spotted owlet	*Athene brama*	2	0	0
Indian silver bill	*Lonchura malabarica*	2	1	50
Scaly‐breasted munia	*Lonchura punctulata*	5	1	20
Common babbler	*Turdoides caudatus*	5	0	0
Rufous treepie	*Dendrocita vagabunda*	1	0	0
Grey francolin	*Francolinus pondicerianus*	1	0	0
Long tailed shrike	*Lanius schach*	6	1	16.7
Oriental magpie‐robin	*Copsychus saularis*	2	0	0
Common chiffchaff	*Phylloscopus collybita*	4	0	0
Lesser white throat	*Sylvia curruca*	7	2	28.6
Yellow wagtail	*Motacilla flava*	7	1	14.3
Blue throat	*Luscinia svecica*	2	0	0
White wagtail	*Motacilla alba*	1	0	0
Crow pheasant	*Centropus sinensis*	1	0	0
Baya weaver	*Ploceus philippinus*	20	1	5
Yellow‐eyed babbler	*Chrysomma sinense*	6	0	0
Streak‐throated swallow	*Petrochelidon fluvicola*	4	0	0
Brahminy starling	*Sturnia pagodarum*	7	0	0
Pied bush chat	*Saxicola caprata*	5	0	0
Black drongo	*Dicrurus macrocercus*	7	1	14.3
Shikra	*Accipiter badius*	2	0	0
Red‐wattled Lapwing	*Vanellus indicus*	2	1	50
Eurasian collared dove	*Streptopelai decaocto*	4	1	25
Green bee‐eater	*Merops orientalis*	5	0	0
White‐throat kingfisher	*Halcyon smyrnensis*	2	0	0
Bank myna	*Acridotheres ginginianus*	3	0	0
Purple sunbird	*Cinnyris asiaticus*	1	0	0

**TABLE 2:** Sample site GPS and toxoplasmosis seroprevalence rates by location in Lahore.

Location	Latitude	Longitude	Total examined	Infected	Prevalence (%)	Chi‐square value	*p* Value
A Block Central Park Lahore	31.326734	74.380912	8	0	0	151.11	**0.011**
Gajju Matah Lahore	31.385342	74.369688	4	1	25		
Ali Raza Abad Lahore	31.437961	74.234039	6	0	0		
Valancia by‐pass Road Lahore	31.397386	74.244431	5	1	20		
Nishter Colony Lahore	31.419670	74.369733	10	1	10		
Gaga Village Lahore	31.432272	74.517334	5	0	0		
Mohlanwal Lahore	31.410889	74.154472	4	1	25		
Maraka Village Lahore	31.374109	74.147324	10	0	0		
Gulshan Yaseen Colony Lahore	31.440290	74.368664	10	2	20		
Mandhila Village Lahore	31.653264	74.499582	17	1	5.9		
Niazi Bhaini Road Lahore	31.648923	74.471406	3	0	0		
Islamia Park Lahore	31.548593	74.307260	11	0	0		
UVAS Lahore	31.574289	74.300879	4	1	25		
Nainsukh Lahore	—	—	5	0	0		
Awanpar Near Ravi River Lahore	31.670776	74.419012	8	0	0		
Ahlu Road Lahore	31.378650	74.368561	7	1	14.3		
Cantt Road Lahore	31.612910	74.403205	10	0	0		
Mureedwall Village Lahore	31.495538	74.228457	14	2	14.3		
Ahbab Colony Lahore	31.481492	74.246255	10	2	20		
Khaliq Abad Lahore	31.439723	74.217057	6	2	33.3		
Bhatti Wall Lahore	31.394538	74.187517	3	1	33.3		
Canal Garden Lahore	31.387871	74.171069	5	1	20		
Elite Town Lahore	31.341929	74.371611	6	0	0		
Jallo Park Lahore	31.569273	74.482030	2	1	50		
Al‐Jannat Society Lahore	31.360340	74.380272	3	0	0		

*Note*: Bold value indicates statistical significant.

The study found a higher prevalence of *T. gondii* antibodies in apparently sick birds (16.7%) compared to apparently healthy birds (9.2%). A statistically significant difference in the prevalence of *T*. *gondii* antibodies was observed with respect to the age of birds (*p* = 0.010). Adult birds were found to be more positive (28.6%) compared to juvenile birds (7.7%). In terms of residential status, no positive samples were observed in migrant, wintering, and summer breeding birds. However, *T. gondii* antibodies were detected in 11% of year‐round resident birds (Table 3). Finally, age, location, order and feeding behaviour were identified as potential risk factors for seroprevalence of *T. gondii* in wild birds based on binary logistic regression analysis. The odds ratios and confidence intervals for each factor are provided in Table 4. While other factors such as gender, health status, urbanicity, and residential status of bird were not found as risk factors for seroprevalence of *T*. *gondii* in wild birds of Lahore in Pakistan.

**TABLE 3:** Risk factors for infection of *Toxoplasma gondii* in birds from the wild in Lahore district, Punjab Pakistan based on chi‐square analysis.

Variable	Factors	Sample examined	Positive	Prevalence (%)	Chi‐square	*p* Value
**Gender**	Male	89	12	13.5	2.079	0.213
	Female	87	6	6.9		
**Urbanicity**	Urban	79	7	8.9	0.292	0.627
	Rural	97	11	11.3		
**Health status**	Healthy	152	14	9.2	1.255	0.263
	Sick	24	4	16.7		
**Age**	Adult	21	6	28.6	8.740	0.010
	Juvenile	155	12	7.7		
**Residential status**	Year‐round resident	163	18	11	1.599	0.660
	Passage migrant	4	0	0		
	Wintering	8	0	0		
	Summer breeder	1	0	0		

**TABLE 4:** Binary logistic regression model for analysis of associated risk factors for birds’ toxoplasmosis.

	Binary logistic regression			
		(95% CI)		
Variable	OR	Lower	Upper	*p* Value
Gender	2.409	0.783	7.413	0.125
Age	0.130	0.035	0.481	0.002
Health status	0.650	0.165	2.557	0.537
Location	0.883	0.801	9.73	0.012
Urbanicity	0.789	0.252	2.464	0.683
Residential status	6571.2	0	—	0.998
Order	0.121	0.024	0.343	0.002
Feeding behaviour	0.232	0.014	0.265	0.001

Variable	Factors	Sample examined	Positive	Prevalence (%)	Chi‐square	*p* Value
**Order**	Passeriformes	154	16	10.4	190.507	0.001
	Galliformes	4	0	0		
	Strigiformes	2	0	0		
	Accipitriformes	2	0	0		
	Charadriiformes	2	1	50		
	Columbiformes	4	1	25		
	Coraciiformes	7	0	0		
	Cuculiformes	1	0	0		
**Feeding**	Insectivorous	41	4	9.8	188.466	0.001
	Omnivores	105	9	8.6		
	Herbivores	7	1	14.3		
	Carnivores	21	3	14.3		
	Granivorous	2	1	50		

## 4 Discussion

This study found an overall seroprevalence of toxoplasmosis to be 10.2% in wild birds in Lahore. Previously, a higher percentage (13%) was reported in a previous study conducted in Kasur district of Punjab, Pakistan (Naveed et al. 2019). However, a significantly higher (21%) seropositivity was reported in Spain (Cabezon et al. 2016). In addition, comparatively higher percentage of wild birds (36.5%) were found seropositive for *T. gondii* antibodies in the Portugal region (Lopes et al. 2021). The seroprevalence of this study was higher than the 2.6% prevalence reported in wild birds of Mexico in the Durango region (Esquivel et al. 2011). A possible reason for such variation in the seroprevalence of *T. gondii* results may be due to different environmental settings and context within each country may facilitate towards the transmission of the toxoplasmosis parasite. The bird's species‐wise prevalence of *T. gondii* antibodies was 40% (14/35) in our study, which was comparatively lower than a previous report from the Kasur district (46%) of Pakistan (Naveed et al. 2019). On the other hand, lower prevalence rates were also observed in other countries such as China (8.7%), Israel (4%), Portugal (4.6%), and Brazil (5%) (Lima et al. 2011; Salant et al. 2009; Waap et al. 2014; Yan et al. 2011). Of note, in addition to country context, another reason for a difference from previous studies may be due to the current study's inclusion of a wide variety of wild bird species, whereas some studies only focused on a few bird species. Another factor which may contribute towards the observed variations in seroprevalence rates of *T. gondii* may be differences in sampled habitats. A 50% incidence of *T. gondii* antibodies in Indian silver bills (*Lonchura malabarica*) was consistent with a recent study conducted in Kasur, Punjab, Pakistan (Naveed et al. 2019). The similarity in results may be due to the shared boundary line between Kasur and Lahore, facilitating the movement of birds between the two districts and potentially increasing the risk of infection, because the movement of birds via interconnected sites can contribute to its sylvatic cycle as well as facilitate the transmission of *T. gondii* (Ammar et al. 2021). In the case of Red‐wattled Lapwing (*V. indicus*), 50% were seropositive for *T. gondii* antibodies in the current investigation. Within Northern lapwing (*Vanellus vanellus*), 20% were seen positive in Italy (Dini et al. 2023). A potential reason for higher prevalence in lapwing may be due to the habitat of this bird which mostly lives in mudflats and open land. Within the Lahore region of Pakistan, it is heavily populated by Lapwing birds and there have already been previous reports of *T. gondii* from animals and human population. Lapwing birds likely had a higher prevalence of *T. gondii* due to the Lahore regions having a contaminated environment leading to greater exposure (Ahmad et al. 2012; Nabi et al. 2018).

Of note, 33.3% of serum samples for common myna (*Acridotheres tristis*) were seropositive, but a lower prevalence (18%) was reported from the Kasur region in a previous study (Naveed et al. 2019). Finding a higher rate of seropositivity in common myna may be a direct result of their feeding behaviour, where common myna typically feed on the ground. The comparatively higher prevalence of *T. gondii* antibodies in the serum of the common myna is evidence of possible contamination of the environment with oocysts which has previously been reported (Ajmal et al. 2013). Moreover, the common myna lives in close contact with human settlements and are often eaten by cats which increases the risk of *T. gondii* infection in cats and the human population (Nabi et al. 2018). House crows (*Corvus splendens*) are widespread in Pakistan and show a 20% seropositivity for *T. gondii* antibodies which might be due to their scavenging nature in the current study. In comparison, 42.6% of Israeli crows in Israel while only 3.3% of hooded crows (*Corvus cornix*) tested positive in central Italy (Mancianti et al. 2020; Salant et al. 2013). Further, this variation in seroprevalence might be due to differences in the environmental conditions of these countries as well as the disease‐resistance capability of different crow species.

Roughly 25% of Eurasian collared dove (*Streptopelia decaocto*) were found seropositive for *T. gondii* in the study area. A comparable seroprevalence of collared dove was reported in a previous study conducted in the district Kasur (Naveed et al. 2019). Hunting dove is a popular practice in Pakistan and consumption of undercooked meat of such wild birds can be a potential source of *T. gondii* infection in humans. Insectivorous and herbivorous bird species serve as excellent indicators of environmental contamination with *T. gondii* oocysts due to their ground‐feeding behaviours, which increase the likelihood of infection (Dubey et al. 2021). Our study observed seropositivity for *T. gondii* antibodies in several bird species, including 11.1% in jungle babbler (*Argya striata*), 14.3% in yellow wagtail (*Motacilla flava*), 16.7% in Asian pied starling (*Sturnus contra*), 20% in scaly‐breasted munia (*Lonchura punctulata*), 22.2% in brown rock chat (*Oenanthe fusca*), and 28.6% in lesser whitethroat (*Curruca curruca*). Similar patterns of *T. gondii* antibody presence have been reported in wild birds with different feeding modes, such as granivorous and insectivorous species, in Portugal (Lopes et al. 2021). In this study, the seroprevalence rate for male birds was 13.5% and 6.9% in female birds. This difference was not statistically significant (*p* > 0.05), suggesting that there is no difference in the likelihood males and females birds contracting toxoplasmosis. A previous study from Pakistan found that male birds had a 13% prevalence rate, and females had a maximum prevalence of 12% which closely aligns with our findings (Naveed et al. 2019). In contrast, a study in Northern Portugal found higher prevalence rates in males (46.8%) compared to females (33.8%) (Tidy et al. 2017). A possible reason for the seropositivity of *T. gondii* in both genders being similar of wild birds could be due to the similar eating habits of both males and females birds (Lopes et al. 2011; Naveed et al. 2019). No significant difference in seropositivity between birds in urban and rural regions or among various health statuses was observed, which aligns with earlier research reported by Tidy et al. (2017). Of note, Salant et al. (2009) reported a statistically significant association with urbanicity, with a *p*‐value of 0.012, indicating a different trend than what was observed within our study findings. Apparently sick birds were more frequently found to be infested with *T. gondii* compared to healthy birds, although the difference was not statistically significant. Similarly, a higher prevalence of toxoplasmosis was observed in apparently sick wild birds (50%) versus healthy ones (11%) in the Kasur region. There have been reports from France of dove fatalities linked to *T. gondii* seropositivity (Rigoulet et al. 2014).

Our study highlights that age may have an impact on the prevalence of *T. gondii* in wild birds. A significant difference in *T. gondii* seroprevalence was observed among different age groups, with a 95% confidence interval of 0.035–0.481, an odds ratio of 0.130, and a *p*‐value of 0.002. This study showed a higher seroprevalence in juvenile birds (28.6%) compared to adults (7.7%), contrasting with findings from Pakistan and China, where adult birds exhibited the highest prevalence compared to juveniles (Naveed et al. 2019; Tian et al. 2012). Previous studies suggest that adult birds might be more exposed to various environmental factors such as water, food, kitchen waste, and human waste, which could increase their infection risk (Rezaei et al. 2019). Toxoplasmosis was detected exclusively in non‐migrant birds, with a seroprevalence of 11%. No seropositive birds were found among the passage migratory, wintering, or summer breeding categories. This contrasts with a previous study by Salant et al. (2009) in Israel, which reported seroprevalence rates of 2.2% for summer and 8.4% for winter. However, this study is limited by smaller sample sizes of individual bird species and would require a much larger investigation to better understand species specific and temporal exposure dynamics.

Our study indicates previous contact of *T. gondii* in a diverse range of wild birds which has direct and indirect links with the human population of the study area. Of note, due to including only select bird species which were properly identified, we urge caution in overinterpretation of these findings towards the general wild bird population in Pakistan. Age, location, and bird species were identified as potential risks for the spread of *T. gondii* in these birds. The seropositivity of birds which are used for human consumption after hunting, poses a risk of transmission of this pathogen to humans.

## Author Contributions

**Shahzad Ali**: initial draft writing, conceptualisation, formal analysis, investigation, supervision, and methodology. **Fareeha Imran**: formal analysis, testing, data curation, initial draft writing, reviews and edits, and validation. **Abigail A. Lilak**: formal analysis, data visualisation, original draft writing. **Tooba Latif**: supervision, methodology, investigation, reviews and edits. **Michael E. von Fricken**: initial draft writing, supervision, conceptualisation, reviews and edits. **Muhammad Tayyub**: formal analysis, collections, testing, reviews and edits. **Usama Saeed**: formal analysis, collections, testing, reviews and edits. **Anam Iftikhar**: supervision, conceptualisation, reviews and edits.

## Funding

This work was supported by the Higher Education Commission, Pakistan via Project No. HEC (FD)/2015/4942. The funding body had no role in the design of the study, data collection, analysis, interpretation of data, or in writing the manuscript.

## Ethics Statement

All procedures were conducted in accordance Animal Ethics Committee of the University of Veterinary and Animal Sciences (UVAS), Lahore, Pakistan (Dr. No. 686).

## Conflicts of Interest

No conflicts of interests to declare

## Data Availability

Data is available in table form throughout manuscript; raw data is available upon reasonable request to corresponding authors.
